# Crystal structure of (*E*)-3-({6-[2-(4-chloro­phen­yl)ethen­yl]-3-oxo-2,3-di­hydro­pyridazin-4-yl}meth­yl)pyridin-1-ium chloride dihydrate

**DOI:** 10.1107/S2056989022003346

**Published:** 2022-03-31

**Authors:** Said Daoui, Emine Berrin Çınar, Necmi Dege, Noureddine Benchat, Eiad Saif, Khalid Karrouchi

**Affiliations:** a Laboratory of Applied Chemistry and Environment (LCAE), Faculty of Sciences, Mohamed I University, 60000 Oujda, Morocco; b Department of Physics, Faculty of Arts and Sciences, Ondokuz Mayıs University, Samsun, 55200, Turkey; c Department of Computer and Electronic Engineering, Sana’a Community College, Sana’a, Yemen; d Laboratory of Analytical Chemistry and Bromatology, Faculty of Medicine and Pharmacy, Mohammed V University in Rabat, Morocco

**Keywords:** crystal structure, pyridazine, pyridazinone derivative, hydrogen bonding, Hirshfeld surfaces

## Abstract

In the title compound, C_18_H_15_ClN_3_O^+^·Cl^−^·2H_2_O, three intra­mol­ecular hydrogen bonds are observed, N—H⋯O, O—H⋯Cl and O—H⋯O. In the crystal, mol­ecules are connected by C—H⋯Cl and N—H⋯O hydrogen bonds. Strong C—H⋯Cl, N—H⋯O, O—H⋯Cl and O—H⋯O hydrogen-bonding inter­actions are implied by the Hirshfeld surface analysis, which indicate that H⋯H contacts make the largest contribution to the overall crystal packing at 33.0%.

## Abstract 2

Three intra­mol­ecular hydrogen bonds are observed in the title compound. In the crystal, mol­ecules are connected by C—H⋯Cl and N—H⋯O hydrogen bonds.

## Chemical context

Pyridazine derivatives are an important class of heterocyclic chemicals that exhibit a wide range of biological actions. For example, their biological activity and anti­microbial properties have been researched extensively (Neumann *et al.*, 2018 ▸). As a result, the pyridazine ring can be found in a range of commercial medicinal compounds, including Cadralazine and Hydralazine, Minaprine, Pipofezine and others (Abu-Hashem *et al.*, 2020 ▸). Pyridazine derivatives can be found also in the backbones of several organic light-emitting diodes (OLEDs) (Liu *et al.*, 2017 ▸), organic solar cells (OSCs) (Knall *et al.*, 2021 ▸), chemosensors (Peng *et al.*, 2020 ▸), tri­fluoro­acetic acid (TFA) sensors (Li *et al.*, 2018 ▸), bioconjugates (Bahou *et al.*, 2021 ▸), low carbon steel corrosion inhibitors (Khadiria *et al.*, 2016 ▸), and several other materials. They have also been used as starting materials in organic synthesis (Llona-Minguez *et al.*, 2017 ▸), acyl­ating agents (Kung *et al.*, 2002 ▸), precursors for N-heterocyclic carbenes (NHCs) (Liu *et al.*, 2012 ▸) and metallocarbene precursors. An overview of aryl­glyoxal monohydrates-based one-pot multi-component synthesis of potentially biologically active pyridazines is given by Mousavi (2022 ▸). 
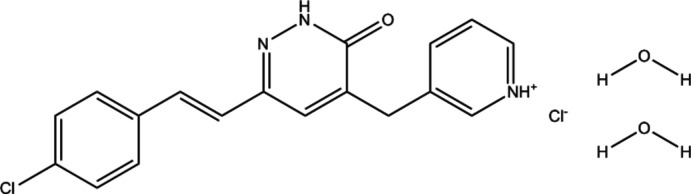


## Structural commentary

**Figure 1:**
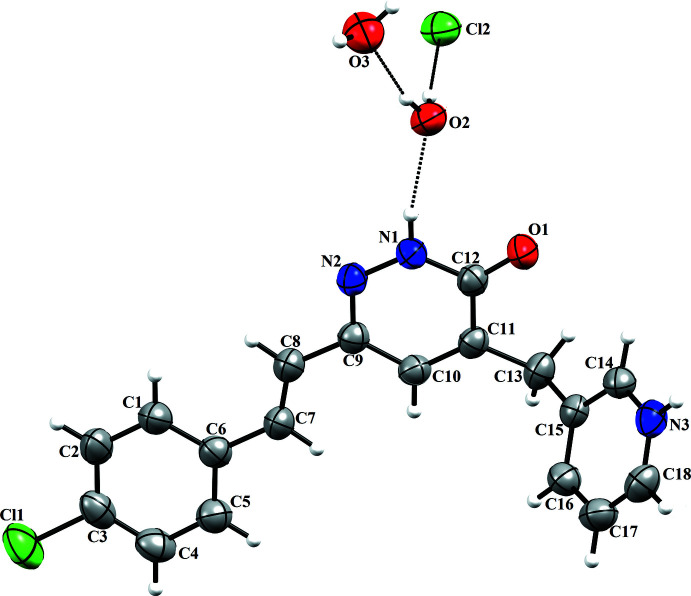
Perspective view and atom labelling of the mol­ecule. Displacement ellipsoids are drawn at the 50% probability level.

A perspective view of the title mol­ecule is shown in Fig. 1 ▸. The pyridazine and pyridine rings subtend a dihedral angle of 57.27 (5)°. The other two rings, pyridazine and chloro­benzene, are almost planar, making an angle of 8.54 (11)°. The lengths of the C=C [1.349 (3) Å], C=N [1.313 (2) Å], N—N [1.351 (2) Å] and C=O [1.237 (2) Å] bonds are comparable with values published for other pyridazinones (see the *Database survey* section). Three intra­mol­ecular hydrogen bonds are observed, N2—H2*C*⋯O2, O2—H2*A*⋯Cl2 and O2—H2*B*⋯O3 (Table 1 ▸).

## Supra­molecular features

**Figure 2:**
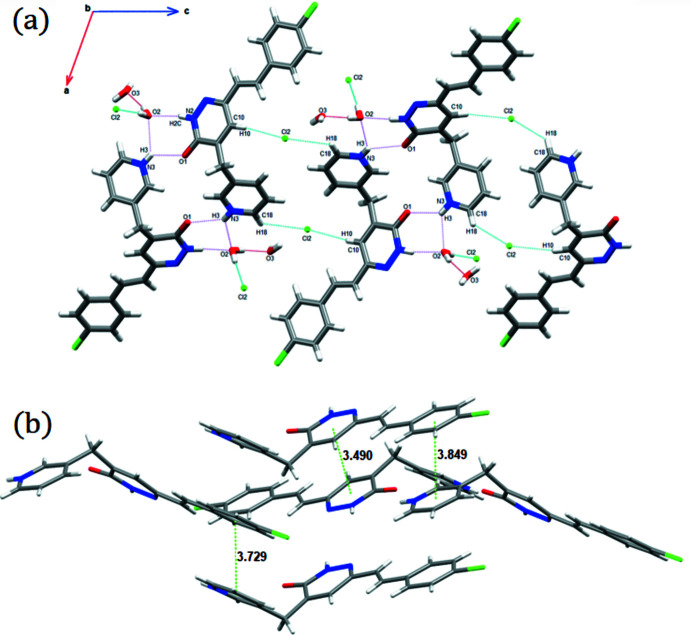
(*a*)View along the *b* axis of the unit cell showing the mol­ecular sheets. (*b*) π–π inter­actions.

The water mol­ecules and chloride anions are located in channels between the organic cations and are connected by O—H⋯O and O—H⋯Cl hydrogen bonds (Table 1 ▸) into chains, which are further connected *via* N—H⋯O and C—H⋯Cl hydrogen bonds into a three-dimensional supra­molecular architecture. Fig. 2 ▸ *a* shows a view of the hydrogen bonds along the *b*-axis direction. π–π inter­actions are present (Fig. 2 ▸ *b*) between the pyridazine rings [centroid–centroid distance = 3.4902 (12) Å], and also between the pyridine and benzene rings [3.7293 (13) and 3.8488 (13) Å], forming sheets.

## Database survey

There are no direct precedents for the structure of the title compound in the crystallographic literature. A search of the Cambridge Structural Database (*ConQuest* version 2021.3.0; Groom *et al.*, 2016 ▸) for the 2,3-di­hydro­pyridazin-4-yl moiety gave various hits, four of them for similar pyridazine compounds but with different substituents on the pyridazine ring: 5-(2-chloro­benz­yl)-6-methyl-3(2*H*)pyridazinone (ZAYJIS; Moreau *et al.*, 1995 ▸), 2-{4-[(5-chloro- 1-benzo­furan-2-yl)meth­yl]-3-methyl-6- oxo-1,6-di­hydro­pyridazin-1-yl}acetate (XULSEE; Boukharsa *et al.*, 2015 ▸) , 4-[3-(tri­fluoro­meth­yl)phen­yl]-5,6,7,8-tetra­hydro­cinnolin-3(2*H*)-one (GISZAK; Wang *et al.*, 2008 ▸) and 5-(2-Chloro­benz­yl)-2-(2-hy­droxy­eth­yl)-6-methyl­pyridazin-3(2*H*)-one (IJEMOZ; Abourichaa *et al.*, 2003 ▸). In ZAYJIS, the lengths of the C=C [1.343 (3) Å], C=N [1.301 (4) Å], N—N [1.357 (3) Å] and C=O [1.255 (3) Å] bonds in the pyridazinone ring are very similar to those in the title compound. In XULSEE, te Cl—C1 bond length is 1.742 (2) Å while in the pyridazine ring, the N1—N2 bond length is 1.365 (2) Å and O2=C2 is 1.228 (2) Å. In GISZAK, the N1—N2 bond is 1.343 (5) Å whereas the C8=O1 bond is 1.246 (5) Å. In IJEMOZ, the pyridazinone ring has a similar value for the N4—N5 bond of 1.367 (2) Å.

## Hirshfeld surface analysis

**Figure 3:**
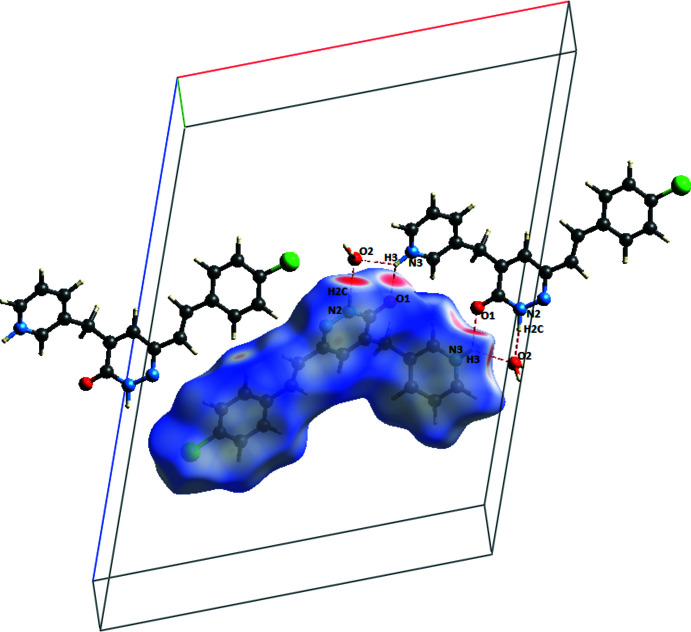
Inter­molecular inter­actions with *d*_norm_ surface.

**Figure 4:**
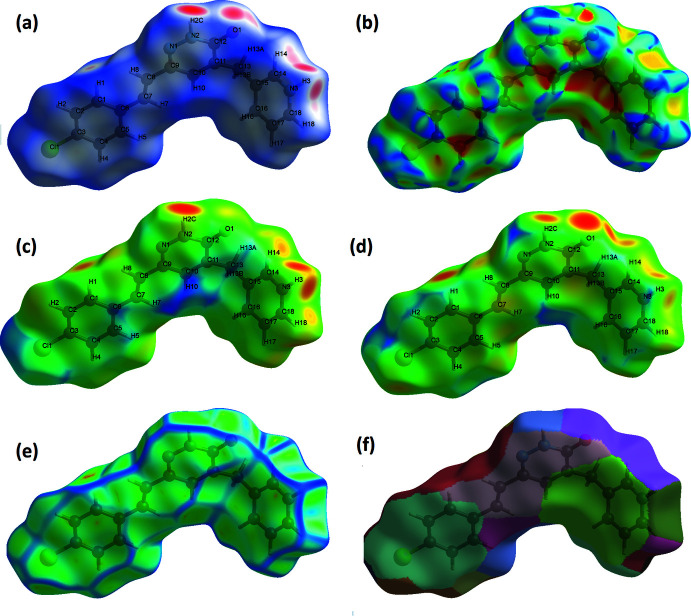
Graphical depictions of the mol­ecular Hirshfeld surfaces; (*a*) *d*_norm_, (*b*)shape-index, (*c*) *d*_i_, (*d*) *d*_e_,(*e*) curvedness and (*f*) fragment-path.

**Figure 5:**
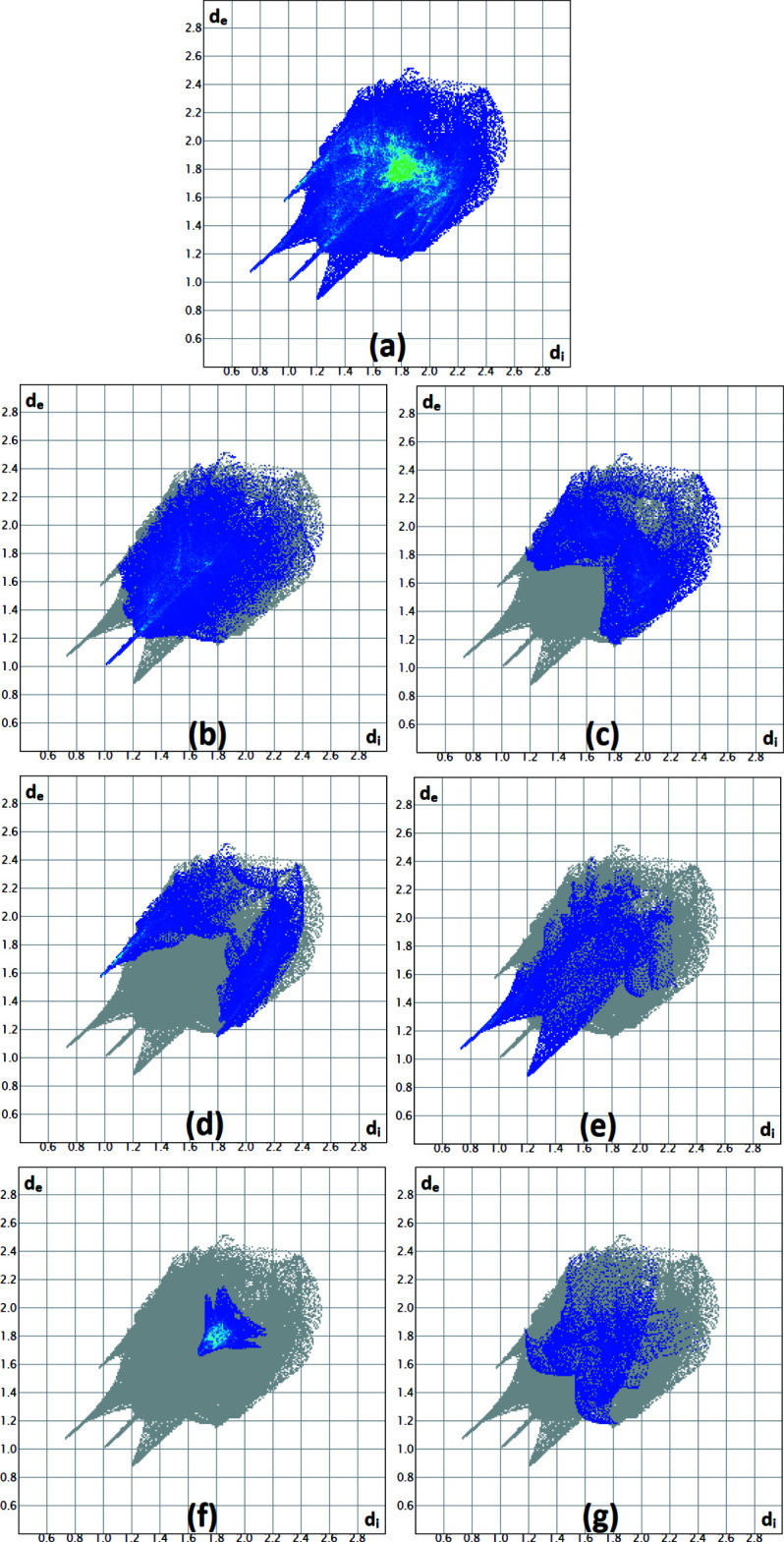
Fingerprint plots of the inter­actions involving the organic cation. (*a*) All contributions and decomposed into the main contributions: (*b*) H⋯H, (*c*) H⋯C/C⋯H, (*d*) H⋯Cl/Cl⋯H, (*e*) H⋯O/O⋯H, (*f*) C⋯C and (*g*) N⋯H/H⋯N inter­actions

**Figure 6:**
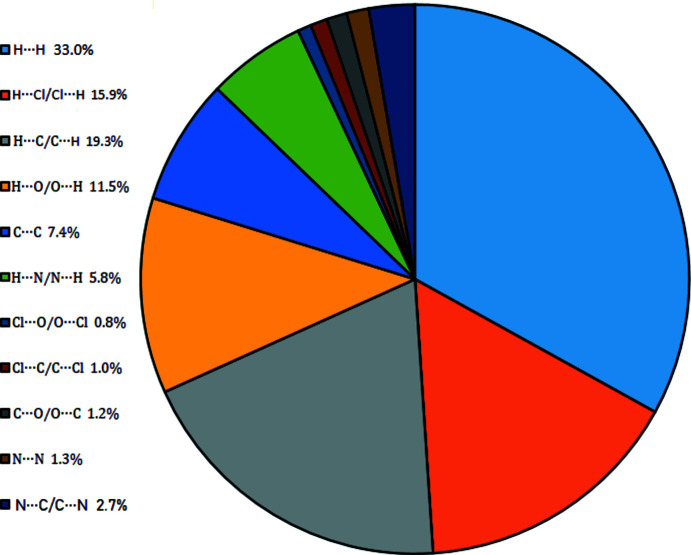
All inter­actions with percentage contributions.

To investigate the effect of the mol­ecular inter­actions on the crystal packing, the Hirshfeld surface (Fig. 3 ▸) and fingerprint plots of the organic cation were analysed (Turner *et al.*, 2017 ▸). In Fig. 4 ▸ *a*, the circular depressions (deep red) on the Hirshfeld surface imply strong hydrogen-bonding inter­actions of types C—H⋯Cl, N—H⋯O, O—H⋯Cl and O—H⋯O. In the shape-index map (Fig. 4 ▸ *b*), the π–π inter­actions are indicated by the red and blue triangles. Fig. 4 ▸ *c* and Fig. 4 ▸ *d* show *d*_i_ and *d*_e_ surfaces and Fig. 4 ▸ *e* and 4*f* the curvedness and fragment path surfaces. Fig. 5 ▸ *a* shows the overall two-dimensional fingerprint plot. The fingerprint plot delineated into H⋯H contacts (33.0% contribution, Fig. 5 ▸ *b*) has a point with the tip at *d*_e_ + *d*_i_ = 2.05 Å. The pair of wings in the fingerprint plot defined into H⋯C/C⋯H contacts (19.3 percent contribution to the HS), Fig.5*c*, has a pair of thin edges at *d*_e_ + *d*_i_ ∼2.99 Å while the pair of wings for the H⋯Cl/Cl⋯H contacts (15.9% contribution, Fig. 5 ▸ *d*) are seen as two spikes with the points at *d*_e_ + *d*_i_ = 2.97 Å and *d*_e_ + *d*_i_ = 2.41 Å. The fingerprint plot for H⋯O/O⋯H contacts (11.5% contribution, Fig. 5 ▸ *e*) has two spikes with the tips at *d*_e_ + *d*_i_ = 2.11 Å and *d*_e_ + *d*_i_ = 1.83 Å. As seen in Fig. 5 ▸ *f* the C⋯C contacts (7.4%) have an arrow-shaped distribution of points with tips at *d*_e_ + *d*_i_ = 3.37 Å. The contributions of the N⋯H/H⋯N contacts to the Hirshfeld surface (5.8%) are less important (Fig. 5 ▸ *g*). Fig. 6 ▸ shows a pie chart of all inter­actions with their percentage contributions.

## Synthesis and crystallization

The title compound was synthesized according to a previously published procedure (Daoui *et al.*, 2019 ▸, 2021 ▸). To a solution of (*E*)-6-(4-chloro­styr­yl)-4,5-di­hydro­pyridazin-3(2*H*)-one (0.23 g, 1 mmol) and nicotinaldehyde (0.107 g, 1 mmol) in 30 ml of ethanol, sodium ethano­ate (0.23 g, 2.8 mmol) was added. The mixture was refluxed for 3 h. The reaction mixture was cooled, diluted with cold water and acidified with concentrated hydro­chloric acid. The precipitate was filtered, washed with water, dried and recrystallized from ethanol. White single crystals were obtained by slow evaporation at room temperature, yield 86%; m.p. 453 K; FT–IR (KBr): ν 3322 (NH), 1651 (C=O), 1584 cm^−1^ (C=N); ^1^H NMR (300 MHz, DMSO-*d*_6_) δ 13.20 (*s*, 1H, H-pyrid­yl) , 8.98 (*d*, *J* = 1.8 Hz, 1H, H-pyrid­yl), 8.83 (*d*, *J* = 5.6 Hz, 1H, H-pyrid­yl), 8.57 (*dt*, *J* = 8.1, 1.8 Hz, 1H, H-pyrid­yl), 8.05 (*s*, 1H, H-pyridazinone) 8.02 (*dd*, *J* = 8.1, 5.6 Hz, 1H, H-pyrid­yl), 7.65 (*d*, *J* = 8.4 Hz, 2H, H1, H-Ar), 7.45 (*d*, *J* = 8.4 Hz, 2H, H 4, H-Ar), 7.36 (*d*, *J* = 16.7 Hz, 1H, CH=CH), 7.08 (,*d J* = 16.7 Hz, 1H, CH=CH), 4.09 ppm (*s*, 2H, CH_2_); ^13^C NMR (75 MHz, DMSO-*d*_6_) δ 160.43, 145.98, 143.89, 141.87, 140.05, 139.25, 137.97, 134.90, 132.84,130.85, 128.82, 128.62, 128.54, 126.80, 125.08, 32.33 ppm. ESI-MS: *m/z* = 324.08 [*M*+H]^+^.

## Refinement details

Crystal data, data collection and structure refinement details are summarized in Table 2 ▸. All C-bound H atoms were placed in calculated positions (C—H = 0.93–0.98 Å) and thereafter treated as riding. A torsional parameter was refined for the methyl group. The positions of N- and O-bound H atoms were refined freely (distances are in Table 1 ▸). For all H atoms, *U*_iso_(H) = 1.2 *U*_eq_(C,N,O).

## Supplementary Material

Crystal structure: contains datablock(s) I. DOI: 10.1107/S2056989022003346/jq2014sup1.cif

Structure factors: contains datablock(s) I. DOI: 10.1107/S2056989022003346/jq2014Isup2.hkl

Supporting information file. DOI: 10.1107/S2056989022003346/jq2014Isup3.cml

CCDC reference: 2161716

Additional supporting information:  crystallographic information; 3D view; checkCIF report

## Figures and Tables

**Table 1:** Hydrogen-bond geometry (Å, °)

*D*—H⋯*A*	*D*—H	H⋯*A*	*D*⋯*A*	*D*—H⋯*A*
C10—H10⋯Cl2^i^	0.93	2.72	3.6387 (19)	168
C18—H18⋯Cl2^ii^	0.93	2.94	3.622 (2)	132
N3—H3⋯O2^iii^	0.80 (3)	2.35 (3)	2.965 (2)	135 (2)
N3—H3⋯O1^iii^	0.80 (3)	2.25 (3)	2.855 (2)	133 (3)
N2—H2*C*⋯O2	0.86 (2)	1.97 (2)	2.801 (2)	161 (2)
O2—H2*A*⋯Cl2	0.83 (2)	2.35 (2)	3.170 (2)	175 (3)
O2—H2*B*⋯O3	0.84 (2)	1.92 (2)	2.739 (3)	167 (3)

Symmetry codes: (i) 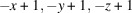 ; (ii) 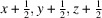 ; (iii) 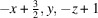 .

**Table 2:** Experimental details

Crystal data	
Chemical formula	C_18_H_15_ClN_3_O^+^·Cl^−^·2H_2_O
*M*_r_	396.26
Crystal system, space group	Monoclinic, *I*2/*a*
Temperature (K)	296
*a*, *b*, *c* (Å)	19.6562 (14), 7.5587 (3), 26.4903 (16)
β (°)	109.762 (5)
*V* (Å^3^)	3704.0 (4)
*Z*	8
Radiation type	Mo *K*α
μ (mm^−1^)	0.37
Crystal size (mm)	0.68 × 0.41 × 0.16

Data collection	
Diffractometer	Stoe IPDS 2
Absorption correction	Numerical (*X-RED32*; Stoe & Cie, 2002 ▸)
*T*_min_, *T*_max_	0.818, 0.961
No. of measured, independent and observed [*I* > 2σ(*I*)] reflections	13762, 5273, 3083
*R*_int_	0.064
(sin θ/λ)_max_ (Å^−1^)	0.702

Refinement	
*R*[*F* ^2^ > 2σ(*F* ^2^)], *wR*(*F* ^2^), *S*	0.050, 0.142, 0.98
No. of reflections	5273
No. of parameters	265
No. of restraints	2
H-atom treatment	H atoms treated by a mixture of independent and constrained refinement
Δρ_max_, Δρ_min_ (e Å^−3^)	0.26, −0.43

Computer programs: *X-AREA* and *X-RED32* (Stoe & Cie, 2002 ▸), *SHELXT2018/3* (Sheldrick, 2015*a* ▸), *OLEX2* (Dolomanov *et al.*, 2009 ▸) and *Mercury* (Macrae *et al.*, 2020 ▸), *WinGX* (Farrugia, 2012 ▸), *SHELXL2018/3* (Sheldrick, 2015*b* ▸), *PLATON* (Spek, 2020 ▸) and *publCIF* (Westrip, 2010 ▸).

## supplementary crystallographic information

### Crystal data

**Table:**

C_18_H_15_ClN_3_O^+^·Cl^−^·2H_2_O	*F*(000) = 1648
*M**_r_* = 396.26	*D*_x_ = 1.421 Mg m^−^^3^
Monoclinic, *I*2/*a*	Mo *K*α radiation, λ = 0.71073 Å
*a* = 19.6562 (14) Å	Cell parameters from 18653 reflections
*b* = 7.5587 (3) Å	θ = 1.6–30.3°
*c* = 26.4903 (16) Å	µ = 0.37 mm^−^^1^
β = 109.762 (5)°	*T* = 296 K
*V* = 3704.0 (4) Å^3^	Prism, colorless
*Z* = 8	0.68 × 0.41 × 0.16 mm

### Data collection

**Table:**

Stoe IPDS 2 diffractometer	5273 independent reflections
Radiation source: sealed X-ray tube, 12 x 0.4 mm long-fine focus	3083 reflections with *I* > 2σ(*I*)
Plane graphite monochromator	*R*_int_ = 0.064
Detector resolution: 6.67 pixels mm^-1^	θ_max_ = 29.9°, θ_min_ = 1.6°
rotation method scans	*h* = −21→27
Absorption correction: numerical (X-RED32; Stoe & Cie, 2002)	*k* = −8→10
*T*_min_ = 0.818, *T*_max_ = 0.961	*l* = −36→36
13762 measured reflections	

### Refinement

**Table:**

Refinement on *F*^2^	Primary atom site location: structure-invariant direct methods
Least-squares matrix: full	Secondary atom site location: difference Fourier map
*R*[*F*^2^ > 2σ(*F*^2^)] = 0.050	Hydrogen site location: mixed
*wR*(*F*^2^) = 0.142	H atoms treated by a mixture of independent and constrained refinement
*S* = 0.98	*w* = 1/[σ^2^(*F*_o_^2^) + (0.0709*P*)^2^] where *P* = (*F*_o_^2^ + 2*F*_c_^2^)/3
5273 reflections	(Δ/σ)_max_ < 0.001
265 parameters	Δρ_max_ = 0.26 e Å^−^^3^
2 restraints	Δρ_min_ = −0.43 e Å^−^^3^

### Special details

**Table:**

Geometry. All esds (except the esd in the dihedral angle between two l.s. planes) are estimated using the full covariance matrix. The cell esds are taken into account individually in the estimation of esds in distances, angles and torsion angles; correlations between esds in cell parameters are only used when they are defined by crystal symmetry. An approximate (isotropic) treatment of cell esds is used for estimating esds involving l.s. planes.

### Fractional atomic coordinates and isotropic or equivalent isotropic displacement parameters (Å^2^)

**Table:**

	*x*	*y*	*z*	*U*_iso_*/*U*_eq_	
Cl2	0.43892 (4)	0.44826 (8)	0.29544 (2)	0.06204 (18)	
Cl1	0.16095 (4)	0.93975 (11)	0.67565 (3)	0.0831 (2)	
O2	0.51631 (9)	0.7860 (3)	0.36086 (6)	0.0569 (4)	
O1	0.63332 (8)	0.6580 (2)	0.47656 (6)	0.0603 (4)	
N2	0.52423 (9)	0.7727 (2)	0.46837 (7)	0.0440 (4)	
N1	0.46811 (9)	0.8166 (2)	0.48443 (6)	0.0437 (4)	
O3	0.47043 (12)	1.0366 (3)	0.28189 (9)	0.0724 (5)	
N3	0.83161 (10)	0.6802 (3)	0.61940 (8)	0.0521 (4)	
C11	0.58620 (10)	0.6148 (3)	0.54755 (7)	0.0414 (4)	
C9	0.47235 (10)	0.7645 (3)	0.53269 (7)	0.0427 (4)	
C12	0.58492 (10)	0.6822 (3)	0.49587 (7)	0.0434 (4)	
C15	0.71539 (10)	0.5767 (3)	0.61025 (7)	0.0420 (4)	
C6	0.34431 (11)	0.8182 (3)	0.61458 (8)	0.0470 (5)	
C10	0.53148 (11)	0.6600 (3)	0.56490 (7)	0.0441 (4)	
H10	0.5323	0.6223	0.5985	0.053*	
C8	0.41189 (11)	0.8140 (3)	0.54971 (8)	0.0477 (5)	
H8	0.3747	0.8785	0.5256	0.057*	
C7	0.40518 (11)	0.7752 (3)	0.59642 (8)	0.0481 (5)	
H7	0.4434	0.7136	0.6206	0.058*	
C14	0.76951 (11)	0.6075 (3)	0.58944 (8)	0.0479 (5)	
H14	0.7626	0.5772	0.5540	0.057*	
C13	0.64570 (11)	0.4898 (3)	0.57732 (8)	0.0496 (5)	
H13A	0.6554	0.4116	0.5515	0.060*	
H13B	0.6288	0.4173	0.6009	0.060*	
C5	0.34973 (12)	0.7804 (3)	0.66698 (9)	0.0540 (5)	
H5	0.3919	0.7288	0.6898	0.065*	
C16	0.72876 (12)	0.6223 (3)	0.66349 (8)	0.0514 (5)	
H16	0.6936	0.6025	0.6792	0.062*	
C18	0.84516 (12)	0.7257 (3)	0.67006 (9)	0.0577 (5)	
H18	0.8892	0.7768	0.6897	0.069*	
C3	0.23208 (13)	0.8927 (3)	0.65221 (9)	0.0566 (6)	
C2	0.22442 (12)	0.9330 (3)	0.60014 (9)	0.0583 (6)	
H2	0.1820	0.9840	0.5776	0.070*	
C1	0.28082 (12)	0.8966 (3)	0.58179 (9)	0.0561 (5)	
H1	0.2762	0.9252	0.5466	0.067*	
C17	0.79392 (13)	0.6969 (3)	0.69313 (9)	0.0593 (6)	
H17	0.8029	0.7274	0.7288	0.071*	
C4	0.29405 (13)	0.8174 (3)	0.68616 (9)	0.0600 (6)	
H4	0.2986	0.7917	0.7215	0.072*	
H3	0.8616 (16)	0.701 (4)	0.6061 (11)	0.070 (8)*	
H2C	0.5201 (13)	0.802 (3)	0.4362 (10)	0.053 (6)*	
H2A	0.4937 (17)	0.700 (3)	0.3444 (12)	0.094 (11)*	
H2B	0.5030 (16)	0.874 (3)	0.3409 (10)	0.079 (9)*	
H3A	0.495 (3)	1.018 (6)	0.2630 (17)	0.127 (16)*	
H3B	0.466 (2)	1.141 (6)	0.2847 (14)	0.095 (13)*	

### Atomic displacement parameters (Å^2^)

**Table:**

	*U*^11^	*U*^22^	*U*^33^	*U*^12^	*U*^13^	*U*^23^
Cl2	0.0694 (4)	0.0648 (4)	0.0496 (3)	0.0006 (3)	0.0170 (2)	0.0021 (2)
Cl1	0.0642 (4)	0.1042 (6)	0.0982 (5)	−0.0103 (4)	0.0502 (4)	−0.0206 (4)
O2	0.0539 (9)	0.0660 (12)	0.0463 (8)	0.0028 (9)	0.0111 (7)	0.0035 (8)
O1	0.0471 (8)	0.0848 (12)	0.0534 (8)	0.0146 (8)	0.0229 (7)	0.0071 (8)
N2	0.0415 (8)	0.0494 (10)	0.0429 (8)	0.0012 (8)	0.0168 (7)	0.0023 (7)
N1	0.0375 (8)	0.0469 (10)	0.0463 (8)	0.0001 (7)	0.0138 (7)	0.0001 (7)
O3	0.0801 (14)	0.0676 (14)	0.0748 (12)	0.0046 (11)	0.0331 (10)	0.0102 (10)
N3	0.0397 (9)	0.0596 (12)	0.0591 (10)	0.0003 (9)	0.0195 (8)	0.0078 (8)
C11	0.0363 (9)	0.0416 (10)	0.0427 (9)	−0.0032 (8)	0.0089 (7)	−0.0017 (7)
C9	0.0394 (9)	0.0448 (11)	0.0431 (9)	−0.0026 (9)	0.0128 (7)	−0.0010 (8)
C12	0.0385 (9)	0.0455 (11)	0.0454 (9)	−0.0018 (8)	0.0130 (8)	−0.0034 (8)
C15	0.0373 (9)	0.0417 (11)	0.0445 (9)	0.0049 (8)	0.0107 (7)	0.0040 (7)
C6	0.0431 (10)	0.0513 (12)	0.0468 (10)	−0.0051 (9)	0.0153 (8)	−0.0065 (8)
C10	0.0424 (10)	0.0486 (12)	0.0396 (9)	−0.0033 (9)	0.0116 (8)	0.0009 (8)
C8	0.0402 (10)	0.0529 (12)	0.0479 (10)	0.0024 (9)	0.0123 (8)	−0.0003 (8)
C7	0.0390 (10)	0.0570 (13)	0.0463 (10)	0.0018 (9)	0.0119 (8)	−0.0015 (8)
C14	0.0458 (11)	0.0560 (12)	0.0423 (9)	0.0039 (10)	0.0154 (8)	0.0037 (8)
C13	0.0397 (10)	0.0481 (12)	0.0552 (10)	0.0008 (9)	0.0085 (9)	0.0019 (9)
C5	0.0495 (11)	0.0632 (14)	0.0496 (11)	−0.0041 (11)	0.0171 (9)	−0.0003 (9)
C16	0.0483 (11)	0.0615 (13)	0.0473 (10)	0.0002 (10)	0.0200 (9)	0.0003 (9)
C18	0.0437 (11)	0.0615 (14)	0.0594 (12)	−0.0045 (10)	0.0062 (9)	0.0012 (10)
C3	0.0494 (11)	0.0620 (14)	0.0662 (13)	−0.0148 (11)	0.0297 (10)	−0.0187 (10)
C2	0.0414 (11)	0.0720 (16)	0.0589 (12)	−0.0006 (11)	0.0133 (9)	−0.0128 (11)
C1	0.0500 (11)	0.0731 (15)	0.0453 (10)	0.0025 (11)	0.0163 (9)	−0.0048 (10)
C17	0.0588 (13)	0.0703 (16)	0.0449 (10)	−0.0028 (12)	0.0125 (10)	−0.0049 (10)
C4	0.0611 (14)	0.0736 (16)	0.0527 (12)	−0.0123 (12)	0.0290 (11)	−0.0046 (10)

### Geometric parameters (Å, º)

**Table:**

Cl1—C3	1.748 (2)	C6—C1	1.389 (3)
O2—H2A	0.825 (18)	C6—C7	1.469 (3)
O2—H2B	0.837 (18)	C10—H10	0.9300
O1—C12	1.237 (2)	C8—C7	1.321 (3)
N2—N1	1.351 (2)	C8—H8	0.9300
N2—C12	1.354 (3)	C7—H7	0.9300
N2—H2C	0.86 (2)	C14—H14	0.9300
N1—C9	1.313 (2)	C13—H13A	0.9700
O3—H3A	0.81 (5)	C13—H13B	0.9700
O3—H3B	0.80 (4)	C5—C4	1.382 (3)
N3—C18	1.322 (3)	C5—H5	0.9300
N3—C14	1.329 (3)	C16—C17	1.376 (3)
N3—H3	0.80 (3)	C16—H16	0.9300
C11—C10	1.349 (3)	C18—C17	1.361 (3)
C11—C12	1.453 (3)	C18—H18	0.9300
C11—C13	1.503 (3)	C3—C4	1.369 (4)
C9—C10	1.426 (3)	C3—C2	1.370 (3)
C9—C8	1.455 (3)	C2—C1	1.380 (3)
C15—C14	1.373 (3)	C2—H2	0.9300
C15—C16	1.388 (3)	C1—H1	0.9300
C15—C13	1.504 (3)	C17—H17	0.9300
C6—C5	1.386 (3)	C4—H4	0.9300
			
H2A—O2—H2B	107 (3)	N3—C14—C15	120.65 (18)
N1—N2—C12	128.25 (16)	N3—C14—H14	119.7
N1—N2—H2C	116.0 (16)	C15—C14—H14	119.7
C12—N2—H2C	115.7 (16)	C11—C13—C15	115.12 (17)
C9—N1—N2	116.31 (16)	C11—C13—H13A	108.5
H3A—O3—H3B	109 (4)	C15—C13—H13A	108.5
C18—N3—C14	122.87 (19)	C11—C13—H13B	108.5
C18—N3—H3	118 (2)	C15—C13—H13B	108.5
C14—N3—H3	119 (2)	H13A—C13—H13B	107.5
C10—C11—C12	118.06 (18)	C4—C5—C6	121.6 (2)
C10—C11—C13	123.32 (18)	C4—C5—H5	119.2
C12—C11—C13	118.51 (17)	C6—C5—H5	119.2
N1—C9—C10	121.28 (17)	C17—C16—C15	120.08 (19)
N1—C9—C8	115.79 (17)	C17—C16—H16	120.0
C10—C9—C8	122.88 (17)	C15—C16—H16	120.0
O1—C12—N2	120.86 (17)	N3—C18—C17	119.2 (2)
O1—C12—C11	124.57 (18)	N3—C18—H18	120.4
N2—C12—C11	114.55 (16)	C17—C18—H18	120.4
C14—C15—C16	117.37 (19)	C4—C3—C2	121.6 (2)
C14—C15—C13	121.23 (17)	C4—C3—Cl1	119.49 (17)
C16—C15—C13	121.36 (18)	C2—C3—Cl1	118.91 (19)
C5—C6—C1	117.58 (18)	C3—C2—C1	118.8 (2)
C5—C6—C7	119.16 (19)	C3—C2—H2	120.6
C1—C6—C7	123.26 (18)	C1—C2—H2	120.6
C11—C10—C9	121.28 (17)	C2—C1—C6	121.6 (2)
C11—C10—H10	119.4	C2—C1—H1	119.2
C9—C10—H10	119.4	C6—C1—H1	119.2
C7—C8—C9	125.74 (19)	C18—C17—C16	119.8 (2)
C7—C8—H8	117.1	C18—C17—H17	120.1
C9—C8—H8	117.1	C16—C17—H17	120.1
C8—C7—C6	127.5 (2)	C3—C4—C5	118.8 (2)
C8—C7—H7	116.3	C3—C4—H4	120.6
C6—C7—H7	116.3	C5—C4—H4	120.6
			
C12—N2—N1—C9	−0.4 (3)	C13—C15—C14—N3	−178.22 (19)
N2—N1—C9—C10	−3.0 (3)	C10—C11—C13—C15	−100.2 (2)
N2—N1—C9—C8	179.47 (17)	C12—C11—C13—C15	83.7 (2)
N1—N2—C12—O1	−177.03 (19)	C14—C15—C13—C11	−92.5 (2)
N1—N2—C12—C11	4.6 (3)	C16—C15—C13—C11	90.2 (2)
C10—C11—C12—O1	176.3 (2)	C1—C6—C5—C4	0.5 (3)
C13—C11—C12—O1	−7.4 (3)	C7—C6—C5—C4	−179.8 (2)
C10—C11—C12—N2	−5.4 (3)	C14—C15—C16—C17	0.6 (3)
C13—C11—C12—N2	170.88 (18)	C13—C15—C16—C17	178.0 (2)
C12—C11—C10—C9	2.6 (3)	C14—N3—C18—C17	0.3 (4)
C13—C11—C10—C9	−173.49 (19)	C4—C3—C2—C1	0.0 (4)
N1—C9—C10—C11	1.8 (3)	Cl1—C3—C2—C1	179.66 (18)
C8—C9—C10—C11	179.15 (19)	C3—C2—C1—C6	0.9 (4)
N1—C9—C8—C7	179.9 (2)	C5—C6—C1—C2	−1.1 (3)
C10—C9—C8—C7	2.5 (3)	C7—C6—C1—C2	179.2 (2)
C9—C8—C7—C6	−178.2 (2)	N3—C18—C17—C16	−0.5 (4)
C5—C6—C7—C8	−174.2 (2)	C15—C16—C17—C18	0.0 (4)
C1—C6—C7—C8	5.4 (4)	C2—C3—C4—C5	−0.5 (4)
C18—N3—C14—C15	0.3 (3)	Cl1—C3—C4—C5	179.77 (19)
C16—C15—C14—N3	−0.8 (3)	C6—C5—C4—C3	0.3 (4)

### Hydrogen-bond geometry (Å, º)

**Table:**

*D*—H···*A*	*D*—H	H···*A*	*D*···*A*	*D*—H···*A*
C10—H10···Cl2^i^	0.93	2.72	3.6387 (19)	168
C18—H18···Cl2^ii^	0.93	2.94	3.622 (2)	132
N3—H3···O2^iii^	0.80 (3)	2.35 (3)	2.965 (2)	135 (2)
N3—H3···O1^iii^	0.80 (3)	2.25 (3)	2.855 (2)	133 (3)
N2—H2*C*···O2	0.86 (2)	1.97 (2)	2.801 (2)	161 (2)
O2—H2*A*···Cl2	0.83 (2)	2.35 (2)	3.170 (2)	175 (3)
O2—H2*B*···O3	0.84 (2)	1.92 (2)	2.739 (3)	167 (3)

Symmetry codes: (i) −*x*+1, −*y*+1, −*z*+1; (ii) *x*+1/2, *y*+1/2, *z*+1/2; (iii) −*x*+3/2, *y*, −*z*+1.
